# 2-[(2-{Bis[2-(2-hy­droxy-5-nitro­benzyl­idene­amino)­eth­yl]amino}­eth­yl)imino­meth­yl]-4-nitro­phenol acetonitrile monosolvate

**DOI:** 10.1107/S1600536810047185

**Published:** 2010-11-20

**Authors:** Kwang Ha

**Affiliations:** a School of Applied Chemical Engineering, The Research Institute of Catalysis, Chonnam National University, Gwangju 500-757, Republic of Korea

## Abstract

In the title compound, C_27_H_27_N_7_O_9_·CH_3_CN, the three nitro groups of the polydentate tripodal Schiff base are located approximately parallel to their respective carrier benzene rings, making dihedral angles of 3.9 (4), 5.0 (4) and 6.3 (4)°. Intra­molecular O—H⋯N hydrogen bonds between the hy­droxy O atoms and the imine N atoms, with O⋯N distances in the range 2.607 (3)–2.665 (3) Å, form nearly planar six-membered rings. In the crystal, weak inter­molecular C—H⋯O and C—H⋯N hydrogen bonds occur and several intra- and inter­molecular π–π inter­actions are present between adjacent benzene rings, with a shortest centroid–centroid distance of 3.507 (2) Å.

## Related literature

For the crystal structure of tris­{2-[(5-bromo­salicyl­idene)amino]­eth­yl}amine, see: Kanesato *et al.* (2001 ▶).
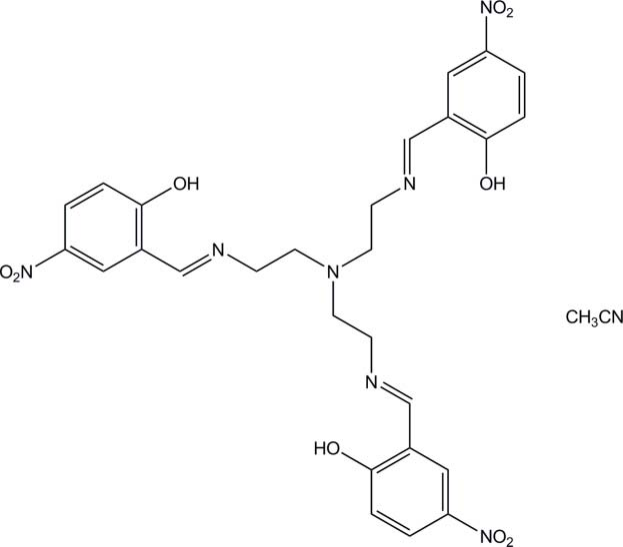


## Experimental

### 

#### Crystal data

C_27_H_27_N_7_O_9_·C_2_H_3_N
*M* *_r_* = 634.61
Triclinic, 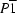

*a* = 10.6097 (9) Å
*b* = 11.8168 (9) Å
*c* = 12.8003 (10) Å
α = 79.054 (2)°
β = 68.293 (2)°
γ = 88.527 (2)°
*V* = 1462.1 (2) Å^3^
*Z* = 2
Mo *K*α radiation
μ = 0.11 mm^−1^
*T* = 200 K
0.32 × 0.13 × 0.11 mm

#### Data collection

Bruker SMART 1000 CCD diffractometer
Absorption correction: multi-scan (*SADABS*; Bruker, 2000 ▶) *T*_min_ = 0.846, *T*_max_ = 0.988
9227 measured reflections
5688 independent reflections
3102 reflections with *I* > 2σ(*I*)
*R*_int_ = 0.038

#### Refinement

*R*[*F* ^2^ > 2σ(*F* ^2^)] = 0.062
*wR*(*F* ^2^) = 0.175
*S* = 1.03
5688 reflections
419 parameters
H-atom parameters constrained
Δρ_max_ = 0.41 e Å^−3^
Δρ_min_ = −0.42 e Å^−3^

### 

Data collection: *SMART* (Bruker, 2000 ▶); cell refinement: *SAINT* (Bruker, 2000 ▶); data reduction: *SAINT*; program(s) used to solve structure: *SHELXS97* (Sheldrick, 2008 ▶); program(s) used to refine structure: *SHELXL97* (Sheldrick, 2008 ▶); molecular graphics: *ORTEP-3* (Farrugia, 1997 ▶) and *PLATON* (Spek, 2009 ▶); software used to prepare material for publication: *SHELXL97*.

## Supplementary Material

Crystal structure: contains datablocks global, I. DOI: 10.1107/S1600536810047185/is2631sup1.cif

Structure factors: contains datablocks I. DOI: 10.1107/S1600536810047185/is2631Isup2.hkl

Additional supplementary materials:  crystallographic information; 3D view; checkCIF report

## Figures and Tables

**Table 1:** Hydrogen-bond geometry (Å, °)

*D*—H⋯*A*	*D*—H	H⋯*A*	*D*⋯*A*	*D*—H⋯*A*
O1—H1*O*⋯N2	0.84	1.87	2.627 (3)	149
O4—H4*O*⋯N4	0.84	1.92	2.665 (3)	147
O7—H7*O*⋯N6	0.84	1.85	2.607 (3)	149
C1—H1*A*⋯N8^i^	0.99	2.56	3.369 (5)	139
C1—H1*B*⋯O4^ii^	0.99	2.41	3.290 (4)	148
C2—H2*B*⋯O2^iii^	0.99	2.44	3.300 (4)	146
C3—H3⋯O7	0.95	2.53	3.297 (4)	138
C6—H6⋯N8^iv^	0.95	2.49	3.360 (5)	153
C9—H9⋯O7	0.95	2.55	3.328 (4)	139
C11—H11*A*⋯O5^v^	0.99	2.40	3.331 (4)	157
C12—H12⋯O5^v^	0.95	2.54	3.339 (4)	142
C16—H16⋯O6^vi^	0.95	2.51	3.330 (4)	145
C25—H25⋯O9^vii^	0.95	2.48	3.359 (4)	153

Symmetry codes: (i) 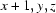; (ii) 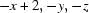; (iii) 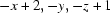; (iv) 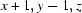; (v) 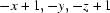; (vi) 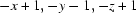; (vii) 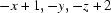.

**Fig. 1.**
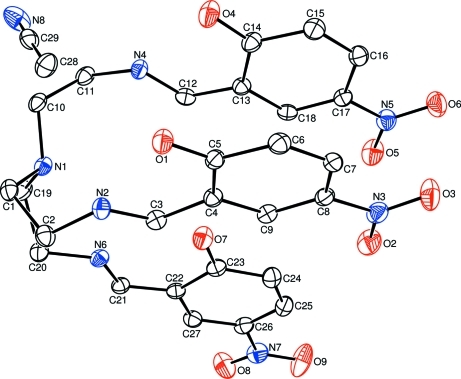
The structure of the title compound, with displacement ellipsoids drawn at the 40% probability level. H atoms are omitted for clarity.

**Fig. 2.**
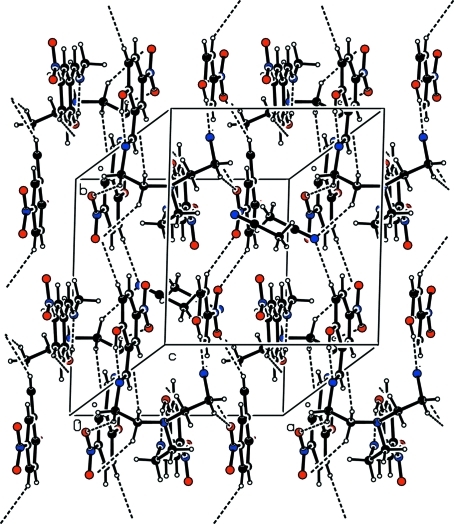
View of the unit-cell contents of the title compound. Hydrogen-bond interactions are drawn with dashed lines.

## supplementary crystallographic information

### Comment

The title compound, C_27_H_27_N_7_O_9_.CH_3_CN, consists of a polydentate tripodal Schiff base and an acetonitrile solvent molecule (Fig. 1). The Schiff base can act as a tribasic hexa- or heptadentate ligand, that is, the N_3_O_3_ or N_4_O_3_ donor atoms can coordinate to a metal ion or metal ions. In the crystal structure, the Schiff base reveals an approximate threefold axis, when viewed down the apical amine N atom (N1) through the plane formed by the atoms C1, C10 and C19, and three nitro groups are located approximately parallel to their respective carrier benzene rings. The N—C bond lengths and the C—N—C bond angles indicate that the apical N1 atom is *sp*^3^-hybridized [*d*(N1—C) = 1.470 (4)–1.480 (4) Å; <C—N1—C = 109.5 (2)–111.7 (2)°] and the other imine N atoms (N2, N4, N6) are *sp*^2^-hybridized [*d*(N═C) = 1.291 (4)–1.307 (4) Å and *d*(N—C) = 1.460 (4)–1.469 (4) Å; <C—N—C = 122.7 (3)–123.9 (3)°]. The compound displays strong intramolecular O—H···N hydrogen bonds between the hydroxy O atoms and the imine N atoms with *d*(O···N) = 2.607 (3)–2.665 (3) Å thus forming a nearly planar six-membered ring (Fig. 2, Table 1). There are also weak intermolecular C—H···O and C—H···N hydrogen bonds with *d*(C···O) = 3.290 (4)–3.359 (4) Å and *d*(C···N) = 3.360 (5)–3.369 (5) Å. Moreover, several intra- and intermolecular π–π interactions between the adjacent benzene rings are present, with a shortest ring centroid-centroid distance of 3.507 (2) Å, and the dihedral angle between the ring planes is 5.1 (2)°.

### Experimental

Tris(2-aminoethyl)amine (0.7305 g, 4.995 mmol) and 5-nitrosalicylaldehyde (2.5077 g, 15.005 mmol) in EtOH (30 ml) were stirred for 3 h at room temperature. The precipitate was then separated by filtration, washed with ether, and dried at 50 °C, to give a yellow powder (2.9135 g). Crystals suitable for X-ray analysis were obtained by slow evaporation from a CH_3_CN solution.

### Refinement

H atoms were positioned geometrically and allowed to ride on their respective parent atoms [C—H = 0.95 Å (CH), 0.99 Å (CH_2_) or 0.98 Å (CH_3_) and O—H = 0.84 Å, and *U*_iso_(H) = 1.2*U*_eq_(C) or 1.5*U*_eq_(methyl C, O)].

### Crystal data

**Table:**

C_27_H_27_N_7_O_9_·C_2_H_3_N	*Z* = 2
*M**_r_* = 634.61	*F*(000) = 664
Triclinic, *P*1	*D*_x_ = 1.441 Mg m^−^^3^
Hall symbol: -P 1	Mo *K*α radiation, λ = 0.71073 Å
*a* = 10.6097 (9) Å	Cell parameters from 2046 reflections
*b* = 11.8168 (9) Å	θ = 2.2–25.2°
*c* = 12.8003 (10) Å	µ = 0.11 mm^−^^1^
α = 79.054 (2)°	*T* = 200 K
β = 68.293 (2)°	Block, yellow
γ = 88.527 (2)°	0.32 × 0.13 × 0.11 mm
*V* = 1462.1 (2) Å^3^	

### Data collection

**Table:**

Bruker SMART 1000 CCD diffractometer	5688 independent reflections
Radiation source: fine-focus sealed tube	3102 reflections with *I* > 2σ(*I*)
graphite	*R*_int_ = 0.038
φ and ω scans	θ_max_ = 26.0°, θ_min_ = 2.1°
Absorption correction: multi-scan (*SADABS*; Bruker, 2000)	*h* = −13→13
*T*_min_ = 0.846, *T*_max_ = 0.988	*k* = −14→13
9227 measured reflections	*l* = −15→13

### Refinement

**Table:**

Refinement on *F*^2^	Primary atom site location: structure-invariant direct methods
Least-squares matrix: full	Secondary atom site location: difference Fourier map
*R*[*F*^2^ > 2σ(*F*^2^)] = 0.062	Hydrogen site location: inferred from neighbouring sites
*wR*(*F*^2^) = 0.175	H-atom parameters constrained
*S* = 1.03	*w* = 1/[σ^2^(*F*_o_^2^) + (0.0681*P*)^2^ + 0.0868*P*] where *P* = (*F*_o_^2^ + 2*F*_c_^2^)/3
5688 reflections	(Δ/σ)_max_ < 0.001
419 parameters	Δρ_max_ = 0.41 e Å^−^^3^
0 restraints	Δρ_min_ = −0.41 e Å^−^^3^

### Special details

**Table:**

Geometry. All e.s.d.'s (except the e.s.d. in the dihedral angle between two l.s. planes) are estimated using the full covariance matrix. The cell e.s.d.'s are taken into account individually in the estimation of e.s.d.'s in distances, angles and torsion angles; correlations between e.s.d.'s in cell parameters are only used when they are defined by crystal symmetry. An approximate (isotropic) treatment of cell e.s.d.'s is used for estimating e.s.d.'s involving l.s. planes.
Refinement. Refinement of *F*^2^ against ALL reflections. The weighted *R*-factor *wR* and goodness of fit *S* are based on *F*^2^, conventional *R*-factors *R* are based on *F*, with *F* set to zero for negative *F*^2^. The threshold expression of *F*^2^ > σ(*F*^2^) is used only for calculating *R*-factors(gt) *etc*. and is not relevant to the choice of reflections for refinement. *R*-factors based on *F*^2^ are statistically about twice as large as those based on *F*, and *R*- factors based on ALL data will be even larger.

### Fractional atomic coordinates and isotropic or equivalent isotropic displacement parameters (Å^2^)

**Table:**

	*x*	*y*	*z*	*U*_iso_*/*U*_eq_	
O1	1.1867 (3)	−0.08637 (18)	0.09431 (18)	0.0448 (6)	
H1O	1.1937	−0.0154	0.0928	0.067*	
O2	0.8123 (3)	−0.2527 (2)	0.6061 (2)	0.0621 (8)	
O3	0.8574 (3)	−0.4164 (2)	0.5510 (2)	0.0703 (8)	
O4	0.8089 (3)	−0.13330 (19)	0.10667 (19)	0.0595 (8)	
H4O	0.7975	−0.0622	0.0925	0.089*	
O5	0.4797 (3)	−0.2368 (2)	0.6310 (2)	0.0608 (8)	
O6	0.4974 (3)	−0.4093 (2)	0.5958 (2)	0.0658 (8)	
O7	0.7816 (3)	0.09156 (19)	0.4737 (2)	0.0502 (7)	
H7O	0.8186	0.1513	0.4252	0.075*	
O8	0.4783 (3)	0.3083 (3)	0.9032 (2)	0.0830 (10)	
O9	0.4522 (3)	0.1281 (3)	0.9805 (2)	0.0913 (11)	
N1	0.9339 (3)	0.2871 (2)	0.1376 (2)	0.0324 (6)	
N2	1.1352 (3)	0.1102 (2)	0.1657 (2)	0.0352 (6)	
N3	0.8708 (3)	−0.3096 (3)	0.5314 (3)	0.0458 (7)	
N4	0.7479 (3)	0.0770 (2)	0.1510 (2)	0.0354 (7)	
N5	0.5246 (3)	−0.3041 (3)	0.5642 (3)	0.0455 (7)	
N6	0.8372 (3)	0.3109 (2)	0.3871 (2)	0.0361 (7)	
N7	0.4978 (3)	0.2066 (3)	0.8974 (3)	0.0566 (9)	
C1	1.0834 (3)	0.3037 (3)	0.0811 (3)	0.0381 (8)	
H1A	1.1088	0.3867	0.0680	0.046*	
H1B	1.1120	0.2813	0.0052	0.046*	
C2	1.1598 (3)	0.2351 (2)	0.1490 (3)	0.0381 (8)	
H2A	1.2583	0.2544	0.1084	0.046*	
H2B	1.1319	0.2575	0.2248	0.046*	
C3	1.0620 (3)	0.0478 (3)	0.2639 (3)	0.0336 (8)	
H3	1.0176	0.0862	0.3256	0.040*	
C4	1.0438 (3)	−0.0738 (3)	0.2849 (3)	0.0308 (7)	
C5	1.1155 (3)	−0.1371 (3)	0.1946 (3)	0.0324 (8)	
C6	1.1040 (3)	−0.2603 (3)	0.2275 (3)	0.0389 (8)	
H6	1.1518	−0.3051	0.1721	0.047*	
C7	1.0271 (3)	−0.3153 (3)	0.3355 (3)	0.0377 (8)	
H7	1.0223	−0.3971	0.3547	0.045*	
C8	0.9544 (3)	−0.2504 (3)	0.4190 (3)	0.0343 (8)	
C9	0.9646 (3)	−0.1321 (3)	0.3945 (3)	0.0323 (7)	
H9	0.9175	−0.0898	0.4524	0.039*	
C10	0.8749 (3)	0.2637 (3)	0.0560 (3)	0.0366 (8)	
H10A	0.9375	0.2169	0.0044	0.044*	
H10B	0.8665	0.3378	0.0083	0.044*	
C11	0.7373 (3)	0.2011 (3)	0.1132 (3)	0.0381 (8)	
H11A	0.6827	0.2347	0.1803	0.046*	
H11B	0.6896	0.2122	0.0589	0.046*	
C12	0.7036 (3)	0.0251 (3)	0.2570 (3)	0.0345 (8)	
H12	0.6720	0.0712	0.3140	0.041*	
C13	0.6991 (3)	−0.0966 (3)	0.2943 (3)	0.0317 (7)	
C14	0.7506 (4)	−0.1716 (3)	0.2118 (3)	0.0388 (8)	
C15	0.7267 (4)	−0.2933 (3)	0.2577 (3)	0.0448 (9)	
H15	0.7619	−0.3456	0.2071	0.054*	
C16	0.6556 (3)	−0.3364 (3)	0.3706 (3)	0.0401 (8)	
H16	0.6389	−0.4173	0.3977	0.048*	
C17	0.6071 (3)	−0.2591 (3)	0.4471 (3)	0.0329 (8)	
C18	0.6295 (3)	−0.1425 (3)	0.4103 (3)	0.0329 (8)	
H18	0.5977	−0.0925	0.4637	0.039*	
C19	0.8723 (4)	0.3882 (3)	0.1849 (3)	0.0386 (8)	
H19A	0.7726	0.3806	0.2069	0.046*	
H19B	0.9062	0.4583	0.1245	0.046*	
C20	0.9027 (4)	0.4034 (3)	0.2889 (3)	0.0394 (8)	
H20A	1.0021	0.4043	0.2694	0.047*	
H20B	0.8703	0.4785	0.3093	0.047*	
C21	0.7671 (3)	0.3281 (3)	0.4904 (3)	0.0359 (8)	
H21	0.7578	0.4052	0.5025	0.043*	
C22	0.7048 (3)	0.2381 (3)	0.5843 (3)	0.0344 (8)	
C23	0.7173 (3)	0.1188 (3)	0.5718 (3)	0.0378 (8)	
C24	0.6567 (3)	0.0327 (3)	0.6723 (3)	0.0436 (9)	
H24	0.6650	−0.0463	0.6666	0.052*	
C25	0.5872 (3)	0.0604 (3)	0.7763 (3)	0.0446 (9)	
H25	0.5484	0.0012	0.8421	0.054*	
C26	0.5731 (3)	0.1773 (3)	0.7858 (3)	0.0419 (9)	
C27	0.6304 (3)	0.2637 (3)	0.6931 (3)	0.0400 (8)	
H27	0.6201	0.3419	0.7018	0.048*	
N8	0.2620 (4)	0.5168 (3)	0.1139 (4)	0.0889 (14)	
C28	0.4516 (4)	0.4093 (4)	0.1626 (4)	0.0712 (13)	
H28A	0.4123	0.3395	0.2205	0.107*	
H28B	0.4975	0.4587	0.1924	0.107*	
H28C	0.5173	0.3877	0.0931	0.107*	
C29	0.3459 (4)	0.4707 (3)	0.1358 (3)	0.0549 (10)	

### Atomic displacement parameters (Å^2^)

**Table:**

	*U*^11^	*U*^22^	*U*^33^	*U*^12^	*U*^13^	*U*^23^
O1	0.0633 (17)	0.0335 (13)	0.0328 (14)	0.0035 (13)	−0.0115 (12)	−0.0087 (11)
O2	0.072 (2)	0.0692 (18)	0.0320 (15)	−0.0026 (15)	−0.0059 (13)	−0.0066 (14)
O3	0.084 (2)	0.0460 (17)	0.0602 (19)	−0.0093 (15)	−0.0123 (16)	0.0112 (15)
O4	0.094 (2)	0.0395 (14)	0.0328 (15)	0.0145 (15)	−0.0087 (14)	−0.0099 (12)
O5	0.0694 (19)	0.0594 (17)	0.0357 (15)	0.0137 (14)	0.0020 (13)	−0.0127 (14)
O6	0.084 (2)	0.0493 (17)	0.0494 (17)	−0.0176 (15)	−0.0115 (15)	0.0005 (14)
O7	0.0633 (18)	0.0406 (14)	0.0415 (15)	0.0102 (13)	−0.0127 (13)	−0.0110 (12)
O8	0.103 (3)	0.076 (2)	0.0479 (18)	−0.0262 (19)	0.0065 (16)	−0.0275 (17)
O9	0.087 (2)	0.101 (2)	0.0378 (17)	0.029 (2)	0.0163 (16)	0.0149 (17)
N1	0.0443 (17)	0.0281 (14)	0.0238 (14)	0.0091 (12)	−0.0097 (13)	−0.0097 (12)
N2	0.0330 (16)	0.0322 (15)	0.0364 (16)	0.0019 (13)	−0.0102 (13)	−0.0029 (13)
N3	0.0440 (19)	0.054 (2)	0.0355 (18)	−0.0034 (16)	−0.0133 (15)	−0.0012 (16)
N4	0.0418 (17)	0.0320 (15)	0.0285 (15)	0.0032 (13)	−0.0096 (13)	−0.0040 (13)
N5	0.0449 (19)	0.0459 (19)	0.0407 (18)	0.0015 (16)	−0.0109 (15)	−0.0072 (16)
N6	0.0416 (17)	0.0404 (16)	0.0278 (15)	0.0046 (13)	−0.0122 (13)	−0.0117 (13)
N7	0.041 (2)	0.080 (3)	0.039 (2)	−0.0021 (19)	−0.0043 (16)	−0.010 (2)
C1	0.046 (2)	0.0312 (18)	0.0274 (18)	−0.0015 (16)	−0.0009 (16)	−0.0082 (15)
C2	0.042 (2)	0.0310 (18)	0.039 (2)	0.0011 (16)	−0.0113 (17)	−0.0085 (16)
C3	0.0307 (19)	0.0388 (19)	0.0314 (18)	0.0094 (15)	−0.0090 (15)	−0.0130 (16)
C4	0.0327 (19)	0.0322 (18)	0.0291 (18)	0.0045 (15)	−0.0124 (15)	−0.0086 (15)
C5	0.0358 (19)	0.0339 (18)	0.0292 (19)	0.0031 (15)	−0.0150 (16)	−0.0044 (16)
C6	0.045 (2)	0.0383 (19)	0.038 (2)	0.0089 (17)	−0.0175 (17)	−0.0149 (17)
C7	0.050 (2)	0.0295 (17)	0.042 (2)	0.0021 (16)	−0.0262 (19)	−0.0080 (16)
C8	0.0319 (19)	0.042 (2)	0.0293 (18)	−0.0027 (16)	−0.0135 (15)	−0.0030 (16)
C9	0.0291 (18)	0.0388 (19)	0.0294 (18)	0.0076 (15)	−0.0106 (15)	−0.0087 (15)
C10	0.050 (2)	0.0351 (18)	0.0230 (17)	0.0054 (16)	−0.0114 (16)	−0.0071 (15)
C11	0.048 (2)	0.0372 (19)	0.0299 (18)	0.0109 (17)	−0.0148 (17)	−0.0092 (16)
C12	0.0306 (19)	0.041 (2)	0.036 (2)	0.0067 (15)	−0.0126 (16)	−0.0181 (17)
C13	0.0313 (19)	0.0330 (18)	0.0311 (18)	0.0050 (15)	−0.0101 (15)	−0.0106 (15)
C14	0.045 (2)	0.040 (2)	0.032 (2)	0.0098 (17)	−0.0143 (17)	−0.0088 (17)
C15	0.059 (2)	0.038 (2)	0.038 (2)	0.0122 (18)	−0.0147 (19)	−0.0171 (17)
C16	0.045 (2)	0.0348 (19)	0.043 (2)	0.0058 (16)	−0.0190 (18)	−0.0093 (17)
C17	0.0287 (19)	0.0395 (19)	0.0290 (18)	0.0020 (15)	−0.0091 (15)	−0.0068 (16)
C18	0.0311 (19)	0.0385 (19)	0.0328 (19)	0.0054 (15)	−0.0123 (15)	−0.0152 (16)
C19	0.057 (2)	0.0314 (18)	0.0251 (18)	0.0093 (16)	−0.0133 (16)	−0.0057 (15)
C20	0.050 (2)	0.0349 (19)	0.0307 (19)	0.0044 (17)	−0.0093 (16)	−0.0114 (16)
C21	0.040 (2)	0.0373 (19)	0.034 (2)	0.0062 (16)	−0.0170 (17)	−0.0110 (16)
C22	0.0310 (19)	0.041 (2)	0.036 (2)	0.0057 (15)	−0.0157 (16)	−0.0123 (17)
C23	0.0314 (19)	0.046 (2)	0.039 (2)	0.0077 (16)	−0.0170 (17)	−0.0101 (18)
C24	0.037 (2)	0.042 (2)	0.050 (2)	0.0075 (17)	−0.0165 (18)	−0.0051 (19)
C25	0.030 (2)	0.053 (2)	0.044 (2)	0.0003 (17)	−0.0125 (17)	0.0064 (19)
C26	0.032 (2)	0.061 (2)	0.032 (2)	0.0016 (18)	−0.0102 (16)	−0.0096 (18)
C27	0.037 (2)	0.047 (2)	0.037 (2)	−0.0007 (17)	−0.0115 (17)	−0.0134 (18)
N8	0.091 (3)	0.051 (2)	0.151 (4)	0.020 (2)	−0.069 (3)	−0.033 (2)
C28	0.069 (3)	0.075 (3)	0.066 (3)	0.011 (3)	−0.029 (2)	0.000 (2)
C29	0.063 (3)	0.039 (2)	0.063 (3)	0.003 (2)	−0.022 (2)	−0.014 (2)

### Geometric parameters (Å, °)

**Table:**

O1—C5	1.262 (4)	C9—H9	0.9500
O1—H1O	0.8400	C10—C11	1.507 (4)
O2—N3	1.239 (3)	C10—H10A	0.9900
O3—N3	1.240 (4)	C10—H10B	0.9900
O4—C14	1.252 (4)	C11—H11A	0.9900
O4—H4O	0.8400	C11—H11B	0.9900
O5—N5	1.236 (3)	C12—C13	1.422 (4)
O6—N5	1.238 (3)	C12—H12	0.9500
O7—C23	1.286 (4)	C13—C18	1.396 (4)
O7—H7O	0.8400	C13—C14	1.450 (4)
O8—N7	1.227 (4)	C14—C15	1.436 (4)
O9—N7	1.222 (4)	C15—C16	1.361 (4)
N1—C10	1.470 (4)	C15—H15	0.9500
N1—C19	1.471 (4)	C16—C17	1.414 (4)
N1—C1	1.480 (4)	C16—H16	0.9500
N2—C3	1.297 (4)	C17—C18	1.366 (4)
N2—C2	1.466 (4)	C18—H18	0.9500
N3—C8	1.432 (4)	C19—C20	1.523 (4)
N4—C12	1.291 (4)	C19—H19A	0.9900
N4—C11	1.469 (4)	C19—H19B	0.9900
N5—C17	1.431 (4)	C20—H20A	0.9900
N6—C21	1.307 (4)	C20—H20B	0.9900
N6—C20	1.460 (4)	C21—C22	1.406 (4)
N7—C26	1.458 (4)	C21—H21	0.9500
C1—C2	1.514 (4)	C22—C27	1.410 (4)
C1—H1A	0.9900	C22—C23	1.446 (4)
C1—H1B	0.9900	C23—C24	1.424 (5)
C2—H2A	0.9900	C24—C25	1.359 (5)
C2—H2B	0.9900	C24—H24	0.9500
C3—C4	1.416 (4)	C25—C26	1.410 (5)
C3—H3	0.9500	C25—H25	0.9500
C4—C9	1.393 (4)	C26—C27	1.364 (4)
C4—C5	1.457 (4)	C27—H27	0.9500
C5—C6	1.432 (4)	N8—C29	1.126 (5)
C6—C7	1.360 (4)	C28—C29	1.432 (5)
C6—H6	0.9500	C28—H28A	0.9800
C7—C8	1.413 (4)	C28—H28B	0.9800
C7—H7	0.9500	C28—H28C	0.9800
C8—C9	1.372 (4)		
			
C5—O1—H1O	109.5	H11A—C11—H11B	107.9
C14—O4—H4O	109.5	N4—C12—C13	124.4 (3)
C23—O7—H7O	109.5	N4—C12—H12	117.8
C10—N1—C19	109.5 (2)	C13—C12—H12	117.8
C10—N1—C1	110.2 (2)	C18—C13—C12	118.3 (3)
C19—N1—C1	111.7 (2)	C18—C13—C14	120.7 (3)
C3—N2—C2	122.7 (3)	C12—C13—C14	120.6 (3)
O2—N3—O3	121.7 (3)	O4—C14—C15	121.7 (3)
O2—N3—C8	119.1 (3)	O4—C14—C13	122.4 (3)
O3—N3—C8	119.2 (3)	C15—C14—C13	115.9 (3)
C12—N4—C11	123.9 (3)	C16—C15—C14	122.6 (3)
O5—N5—O6	121.5 (3)	C16—C15—H15	118.7
O5—N5—C17	119.3 (3)	C14—C15—H15	118.7
O6—N5—C17	119.2 (3)	C15—C16—C17	119.0 (3)
C21—N6—C20	123.8 (3)	C15—C16—H16	120.5
O9—N7—O8	122.7 (3)	C17—C16—H16	120.5
O9—N7—C26	118.5 (4)	C18—C17—C16	121.7 (3)
O8—N7—C26	118.8 (3)	C18—C17—N5	119.3 (3)
N1—C1—C2	113.8 (3)	C16—C17—N5	118.9 (3)
N1—C1—H1A	108.8	C17—C18—C13	120.0 (3)
C2—C1—H1A	108.8	C17—C18—H18	120.0
N1—C1—H1B	108.8	C13—C18—H18	120.0
C2—C1—H1B	108.8	N1—C19—C20	113.4 (3)
H1A—C1—H1B	107.7	N1—C19—H19A	108.9
N2—C2—C1	112.5 (3)	C20—C19—H19A	108.9
N2—C2—H2A	109.1	N1—C19—H19B	108.9
C1—C2—H2A	109.1	C20—C19—H19B	108.9
N2—C2—H2B	109.1	H19A—C19—H19B	107.7
C1—C2—H2B	109.1	N6—C20—C19	111.5 (3)
H2A—C2—H2B	107.8	N6—C20—H20A	109.3
N2—C3—C4	124.2 (3)	C19—C20—H20A	109.3
N2—C3—H3	117.9	N6—C20—H20B	109.3
C4—C3—H3	117.9	C19—C20—H20B	109.3
C9—C4—C3	119.2 (3)	H20A—C20—H20B	108.0
C9—C4—C5	120.8 (3)	N6—C21—C22	123.3 (3)
C3—C4—C5	119.9 (3)	N6—C21—H21	118.4
O1—C5—C6	122.0 (3)	C22—C21—H21	118.4
O1—C5—C4	122.0 (3)	C21—C22—C27	119.9 (3)
C6—C5—C4	116.0 (3)	C21—C22—C23	121.0 (3)
C7—C6—C5	122.2 (3)	C27—C22—C23	119.1 (3)
C7—C6—H6	118.9	O7—C23—C24	121.3 (3)
C5—C6—H6	118.9	O7—C23—C22	121.2 (3)
C6—C7—C8	119.9 (3)	C24—C23—C22	117.5 (3)
C6—C7—H7	120.0	C25—C24—C23	121.9 (3)
C8—C7—H7	120.0	C25—C24—H24	119.1
C9—C8—C7	121.0 (3)	C23—C24—H24	119.1
C9—C8—N3	119.8 (3)	C24—C25—C26	119.6 (3)
C7—C8—N3	119.3 (3)	C24—C25—H25	120.2
C8—C9—C4	120.1 (3)	C26—C25—H25	120.2
C8—C9—H9	119.9	C27—C26—C25	121.3 (3)
C4—C9—H9	119.9	C27—C26—N7	119.3 (3)
N1—C10—C11	113.3 (3)	C25—C26—N7	119.5 (3)
N1—C10—H10A	108.9	C26—C27—C22	120.6 (3)
C11—C10—H10A	108.9	C26—C27—H27	119.7
N1—C10—H10B	108.9	C22—C27—H27	119.7
C11—C10—H10B	108.9	C29—C28—H28A	109.5
H10A—C10—H10B	107.7	C29—C28—H28B	109.5
N4—C11—C10	111.9 (3)	H28A—C28—H28B	109.5
N4—C11—H11A	109.2	C29—C28—H28C	109.5
C10—C11—H11A	109.2	H28A—C28—H28C	109.5
N4—C11—H11B	109.2	H28B—C28—H28C	109.5
C10—C11—H11B	109.2	N8—C29—C28	178.4 (4)
			
C10—N1—C1—C2	−131.8 (3)	C13—C14—C15—C16	2.5 (5)
C19—N1—C1—C2	106.1 (3)	C14—C15—C16—C17	−2.2 (5)
C3—N2—C2—C1	−107.8 (3)	C15—C16—C17—C18	0.1 (5)
N1—C1—C2—N2	62.4 (3)	C15—C16—C17—N5	175.8 (3)
C2—N2—C3—C4	−175.4 (3)	O5—N5—C17—C18	−2.8 (4)
N2—C3—C4—C9	178.9 (3)	O6—N5—C17—C18	175.5 (3)
N2—C3—C4—C5	3.2 (5)	O5—N5—C17—C16	−178.6 (3)
C9—C4—C5—O1	179.2 (3)	O6—N5—C17—C16	−0.3 (4)
C3—C4—C5—O1	−5.1 (5)	C16—C17—C18—C13	1.6 (5)
C9—C4—C5—C6	−2.9 (4)	N5—C17—C18—C13	−174.1 (3)
C3—C4—C5—C6	172.7 (3)	C12—C13—C18—C17	171.2 (3)
O1—C5—C6—C7	−179.9 (3)	C14—C13—C18—C17	−1.2 (4)
C4—C5—C6—C7	2.3 (4)	C10—N1—C19—C20	167.1 (3)
C5—C6—C7—C8	0.5 (5)	C1—N1—C19—C20	−70.5 (3)
C6—C7—C8—C9	−2.8 (5)	C21—N6—C20—C19	−132.3 (3)
C6—C7—C8—N3	178.3 (3)	N1—C19—C20—N6	−67.3 (4)
O2—N3—C8—C9	−2.3 (4)	C20—N6—C21—C22	−179.8 (3)
O3—N3—C8—C9	176.7 (3)	N6—C21—C22—C27	−179.8 (3)
O2—N3—C8—C7	176.6 (3)	N6—C21—C22—C23	1.2 (5)
O3—N3—C8—C7	−4.5 (4)	C21—C22—C23—O7	−2.5 (5)
C7—C8—C9—C4	2.2 (4)	C27—C22—C23—O7	178.4 (3)
N3—C8—C9—C4	−179.0 (3)	C21—C22—C23—C24	177.0 (3)
C3—C4—C9—C8	−174.9 (3)	C27—C22—C23—C24	−2.0 (4)
C5—C4—C9—C8	0.8 (4)	O7—C23—C24—C25	−179.3 (3)
C19—N1—C10—C11	−79.5 (3)	C22—C23—C24—C25	1.2 (5)
C1—N1—C10—C11	157.2 (2)	C23—C24—C25—C26	0.5 (5)
C12—N4—C11—C10	114.6 (3)	C24—C25—C26—C27	−1.5 (5)
N1—C10—C11—N4	−78.8 (3)	C24—C25—C26—N7	179.6 (3)
C11—N4—C12—C13	172.5 (3)	O9—N7—C26—C27	−176.8 (3)
N4—C12—C13—C18	−170.1 (3)	O8—N7—C26—C27	6.0 (5)
N4—C12—C13—C14	2.4 (5)	O9—N7—C26—C25	2.1 (5)
C18—C13—C14—O4	177.3 (3)	O8—N7—C26—C25	−175.1 (3)
C12—C13—C14—O4	5.0 (5)	C25—C26—C27—C22	0.6 (5)
C18—C13—C14—C15	−0.8 (4)	N7—C26—C27—C22	179.5 (3)
C12—C13—C14—C15	−173.0 (3)	C21—C22—C27—C26	−177.9 (3)
O4—C14—C15—C16	−175.5 (3)	C23—C22—C27—C26	1.2 (5)

### Hydrogen-bond geometry (Å, °)

**Table:**

*D*—H···*A*	*D*—H	H···*A*	*D*···*A*	*D*—H···*A*
O1—H1O···N2	0.84	1.87	2.627 (3)	149
O4—H4O···N4	0.84	1.92	2.665 (3)	147
O7—H7O···N6	0.84	1.85	2.607 (3)	149
C1—H1A···N8^i^	0.99	2.56	3.369 (5)	139
C1—H1B···O4^ii^	0.99	2.41	3.290 (4)	148
C2—H2B···O2^iii^	0.99	2.44	3.300 (4)	146
C3—H3···O7	0.95	2.53	3.297 (4)	138
C6—H6···N8^iv^	0.95	2.49	3.360 (5)	153
C9—H9···O7	0.95	2.55	3.328 (4)	139
C11—H11A···O5^v^	0.99	2.40	3.331 (4)	157
C12—H12···O5^v^	0.95	2.54	3.339 (4)	142
C16—H16···O6^vi^	0.95	2.51	3.330 (4)	145
C25—H25···O9^vii^	0.95	2.48	3.359 (4)	153

Symmetry codes: (i) *x*+1, *y*, *z*; (ii) −*x*+2, −*y*, −*z*; (iii) −*x*+2, −*y*, −*z*+1; (iv) *x*+1, *y*−1, *z*; (v) −*x*+1, −*y*, −*z*+1; (vi) −*x*+1, −*y*−1, −*z*+1; (vii) −*x*+1, −*y*, −*z*+2.
